# Loss of type I interferon signaling in Kupffer cells triggers viral spread and fatal liver damage in a mouse model of Crimean-Congo hemorrhagic fever

**DOI:** 10.1371/journal.ppat.1014511

**Published:** 2026-08-18

**Authors:** Shintaro Yamada, Masayuki Shimojima, Takeshi Saito, Junki Maruyama, Hiroki Kato

**Affiliations:** 1 Institute of Cardiovascular Immunology, University Hospital Bonn, University of Bonn, Bonn, Germany; 2 Department of Virology I, National Institute of Infectious Diseases, Japan Institute for Health Security, Musashimurayama, Tokyo, Japan; 3 Department of Pathology, The University of Texas Medical Branch at Galveston, Galveston, Texas, United States of America; 4 Institute for Human Infections and Immunity, The University of Texas Medical Branch at Galveston, Galveston, Texas, United States of America; University of Pittsburgh, UNITED STATES OF AMERICA

## Abstract

Crimean-Congo hemorrhagic fever (CCHF), which is caused by infection with the CCHF virus (CCHFV), is the most widespread hemorrhagic infectious disease. In severe cases, liver damage is a salient manifestation. However, the detailed mechanism is not yet fully understood. To investigate the pathogenesis of CCHF-related liver damage, we infected type I interferon receptor 1 knockout (IFNAR1^-/-^) mice with Hazara virus (HAZV), which is a surrogate pathogen of CCHFV, as well as with the CCHFV itself. HAZV infection caused CCHF-like symptoms, including severe liver damage, alongside inflammatory responses. HAZV infection in IFNAR1^-/-^ mice additionally lacking mitochondrial antiviral signaling protein (MAVS) induced minimal cytokine responses; however, these mice still exhibited weight loss and liver damage, albeit with a significantly delayed onset of lethal outcomes. We found that loss of liver-resident macrophage, Kupffer cells, occurred prior to viral spread in hepatocytes and liver damage in these mice. Notably, mice lacking IFNAR1 in Kupffer cells, but not mice lacking IFNAR1 in hepatocytes, also lost Kupffer cell population and exhibited lethal liver damage, following HAZV and CCHFV infection. Our findings indicate that IFNAR1 signaling in mononuclear phagocytes, especially Kupffer cells, is essential for preventing viral spread and fatal liver damage and raise the possibility that inflammatory cytokines- and viral replication-mediated Kupffer cell loss synergistically drives both processes.

## Author summary

CCHF is an infectious disease of worldwide concern. We need to develop specific treatments for CCHF. This study raises the possibility that the loss of Kupffer cells due to CCHFV infection is a causative factor in fatal liver damage and proposes that this could be a new potential therapeutic target against CCHF. Additionally, we are the first to report on the use of myeloid cell lineage-specific IFNAR1-deficient mice as a lethal mouse model of CCHF. Using this model, rather than conventional systemic knockout mice such as IFNAR1^-/-^ mice, our study sheds new light on the pathogenesis of liver damage during CCHFV infection, and on the development of specific CCHF treatments.

## Introduction

Crimean-Congo hemorrhagic fever (CCHF) is a life-threatening infectious disease for humans with a high fatality rate of 10–30% [1,2]. It is distributed worldwide throughout Central Asia, the Middle East, Eastern Europe, Southern Europe, and Africa [3]. The endemic area may be larger than expected or may have expanded in recent decades, as the disease has been reported for the first time in several countries, including Turkey, India and Spain [4–6]. The causative pathogen, CCHF virus (CCHFV), is a trisegmented single-stranded RNA virus belonging to the *Nairoviridae* family within the *Hareavirales* order. CCHFV is transmitted to human through tick bites from infected ticks, or through contacts with the blood or body fluids from infected animals or humans. Currently, there are no licensed vaccines or specific treatments. Ribavirin, an antiviral nucleoside analogue, is the standard medication chosen for CCHF patients; however, its significant benefits need to be discussed.

CCHF disease progression can be divided into four distinct stages: incubation, pre-hemorrhagic, hemorrhagic, and convalescence [2,7,8]. Following the incubation period, the disease progresses to the pre-hemorrhagic stage, exhibiting nonspecific symptoms of febrile illness including fever, headache, nausea, malaise, and myalgia. During the hemorrhagic stage, patients show uncontrolled bleeding, such as petechia, ecchymoses, epistaxis, and melena. They also exhibit inflammatory immune responses and liver damage. In severe cases, disseminated intravascular coagulation, shock, and death may occur. Hematology and blood chemistry tests often reveal thrombocytopenia, increased levels of inflammatory cytokine, and elevated liver enzymes such as alanine aminotransferase (ALT) and aspartate aminotransferase (AST) [9–12]. These factors, in addition to the blood virus loads, are strongly correlated with severe outcomes and might be significant predictors of disease progression and death. Liver damage is a common feature of CCHF, as evidenced by elevated liver enzyme levels [9,10]. Although autopsy reports on CCHF patients are limited, they reveal that the CCHFV antigen is frequently found in the liver and colocalizes with hepatocytes, endothelial cells, and mononuclear phagocytes [6,13,14]. This indicates that the liver is a primary organ for CCHFV replication.

Animal models of infectious diseases are indispensable for developing antivirals and vaccines, and for fully understanding disease pathogenesis. To date, various small laboratory animals, including mice, rabbits, guinea pigs, and hamsters, have been challenged with CCHFV to evaluate susceptibility [15]. While CCHFV infection does not result in disease development in adult immunocompetent rodents, it causes severe disease in immunocompromised rodents, such as mice lacking type I interferon receptor 1 (IFNAR1) [16–19], those lacking both the IFNAR1 and interferon γ receptor [20], those lacking signal transducer and activator of transcription 1 (STAT1) [21], and those with transiently suppressed IFNAR1-signaling following inoculation with anti-IFNAR1 monoclonal antibody [22]. These mice deficient in antiviral immunity exhibit high viremia, inflammatory immune responses, liver damage, and death, as observed in severe human CCHF cases. Notably, a previous study showed that a signaling pathway regulated by mitochondrial antiviral signaling protein (MAVS) is a major source for the inflammatory cytokine production in transiently IFNAR1-blocked mice infected with CCHFV and that deficiency in MAVS improves the survival of them [23]. Furthermore, treatment with a neutralizing antibody against tumor necrosis factor-alpha (TNF-α) alone significantly reduces liver pathology and protects against death in the same mouse model. This is the first important report about the contribution of host immune responses triggered by TNF-α to the CCHF pathogenesis through liver damage. However, the mechanism by which TNF-α expression disturbs liver homeostasis and results in liver damage remains to be elucidated.

CCHFV is highly pathogenic to humans, and there are currently no effective countermeasures against infection. In general, work with CCHFV requires a biosafety level 4 (BSL-4) facility to prevent the virus from spreading in nature, as well as to protect researchers from exposure to it. However, BSL-4 facilities are limited worldwide, and personnel require specific training to work there. These constraints would hinder extensive research utilizing authentic CCHFV. Among the *Nairoviridae* family currently reported, Hazara virus (HAZV) isolated from *Ixodes redikorzevi* ticks in Pakistan, shares the same serogroup with CCHFV. It can be easily handled in a BSL-2 facility as it is nonpathogenic to humans [24]. Therefore, HAZV is widely utilized as a surrogate pathogen of CCHFV. A previous study showed that HAZV infection develops severe disease along with high viremia, liver pathology, and death in IFNAR1 knockout (IFNAR1^-/-^) mice, as observed in CCHFV-infected IFNAR1^-/-^ mice [25]. However, a detailed mechanistic analysis of the mice has yet to be performed.

The aim of this study is to understand the mechanisms underlying fatal liver damage during CCHFV infection. Through analyses of immunodeficient mouse models including systemic and cell type-specific IFNAR1^-/-^ mice, we suggest that Kupffer cell loss mediated by host immune responses and virus replication results in viral spread to hepatocytes, thereby leading to fatal liver damage in CCHFV infection. Our findings provide new insights into the mechanism of liver damage in CCHF.

## Results

### HAZV infection causes severe CCHF-like symptoms in IFNAR1^-/-^ mice

**Fig 1:**
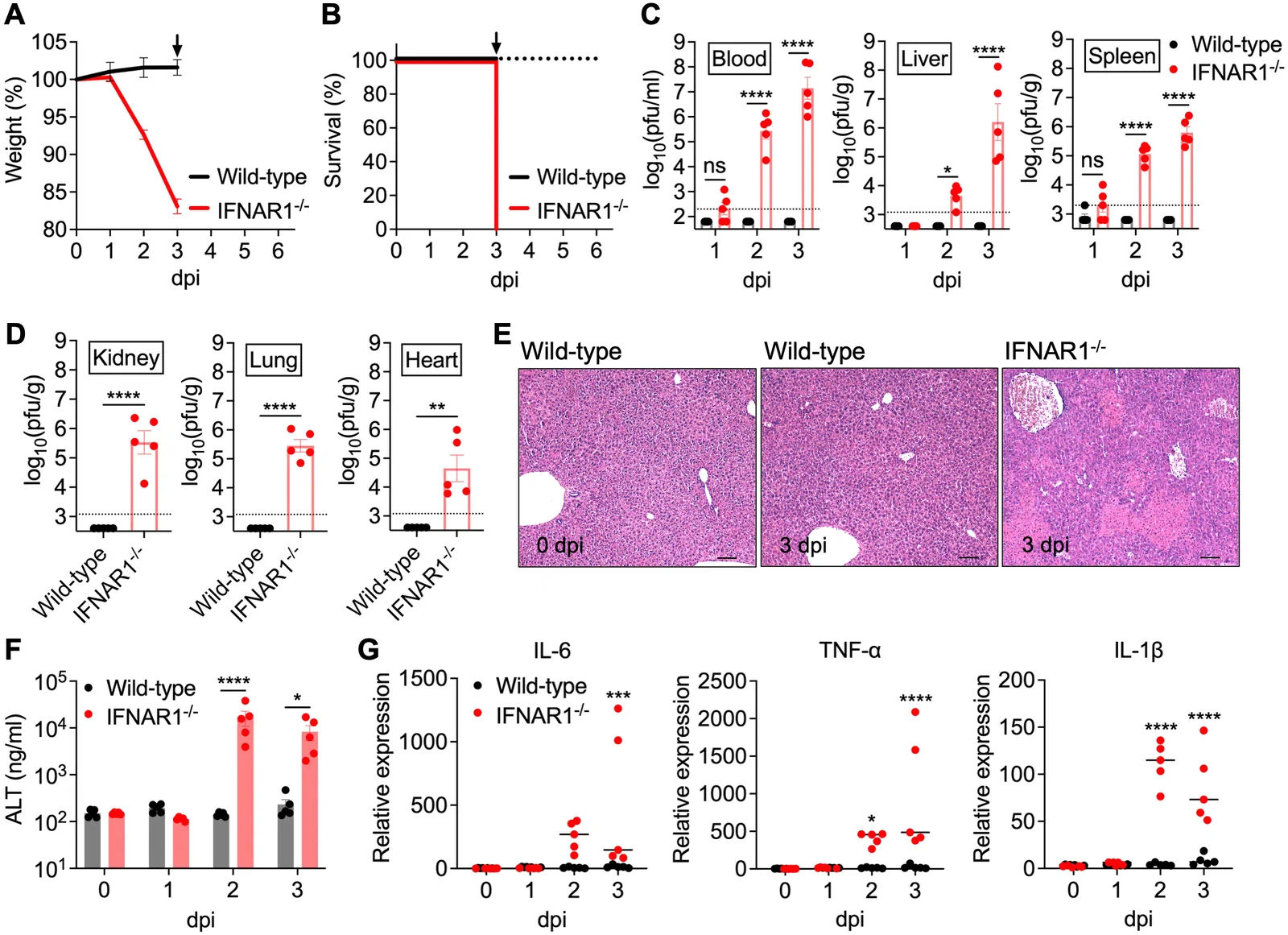
IFNAR1^-/-^ mice exhibit liver damage along with inflammatory responses following HAZV infection. **(A, B)** Weight change (A) or survival (B) of wild-type and IFNAR1^-/-^ mice following HAZV infection (n = 6/each group). Arrows indicate the time point at which mice were euthanized for sampling. **(C, D)** Viral loads of HAZV in the blood, livers, spleens (C), kidneys, lungs and hearts (D) of wild-type and IFNAR1^-/-^ mice at the indicated time points (n = 5/each group). The detection limit is indicated as a dotted line. **(E)** Representative hematoxylin and eosin (H&E) staining images of liver sections from uninfected wild-type mice, and from HAZV-infected wild-type mice and IFNAR1^-/-^ mice at 3 dpi. Scale bars: 100 μm. **(F)** ALT levels in the plasma from wild-type mice and IFNAR1^-/-^ mice at the indicated time points (n = 5/each group). **(G)** qRT-PCR analysis for IL-6, TNF-α, and IL-1β mRNA relative quantities in the livers of wild-type mice and IFNAR1^-/-^ mice at the indicated time points (n = 5/each group). (A-D, F, and G) Data are pooled from two independent experiments and shown as mean ± SEM. The *p*-values were calculated using a two-way ANOVA with Tukey’s multiple comparisons test (C, F, and G) and two-tailed unpaired t test (D). ns, not significant, *p < 0.05, **p < 0.01, ***p < 0.001, ****p < 0.0001.

A previous study shows that HAZV infection causes lethal disease in IFNAR1^-/-^ mice [25]. To investigate the pathogenesis of liver damage caused by CCHFV infection, we analyzed IFNAR1^-/-^ mice following HAZV infection as a surrogate model for CCHFV infection. Consistent with the previous study, infected IFNAR1^-/-^ mice exhibited weight loss and lethality within three days post infection (dpi), while wild-type mice showed no weight loss or clinical symptoms (Fig 1A, 1B). In addition, viral loads in the blood, livers, and spleens of IFNAR1^-/-^ mice were significantly higher at 2 and 3 dpi than those in wild-type mice (Fig 1C). Higher virus loads were also observed in the kidneys, lungs, and hearts of IFNAR1^-/-^ mice (Fig 1D). Histopathological analysis revealed that the livers of infected IFNAR1^-/-^ mice at 3 dpi exhibited extensive hepatocellular necrosis, in contrast to the livers of uninfected and infected wild-type mice (Fig 1E). ALT levels in the plasma of IFNAR1^-/-^ mice, but not wild-type mice, were significantly increased at 2 and 3 dpi (Fig 1F). These results suggest that HAZV infection causes severe liver damage in IFNAR1^-/-^ mice, as has been observed in severe CCHF [17]. To investigate the inflammatory immune response during HAZV infection, we measured the levels of inflammatory cytokine mRNA including interleukin-6 (IL-6), TNF-α, and IL-1β, in the livers (Fig 1G). These inflammatory cytokines were clearly upregulated at 2 and 3 dpi in the livers of IFNAR1^-/-^ mice, whereas there was no obvious increase in cytokine expression in the livers of wild-type mice. These results indicate that HAZV infection causes severe CCHF-like outcomes, including systemic infection, high viremia, liver pathology, and intense inflammation in IFNAR1^-/-^ mice.

### The RIG-I/MAVS pathway is a major source of inflammatory cytokines in response to HAZV infection *in vitro*

**Fig 2:**
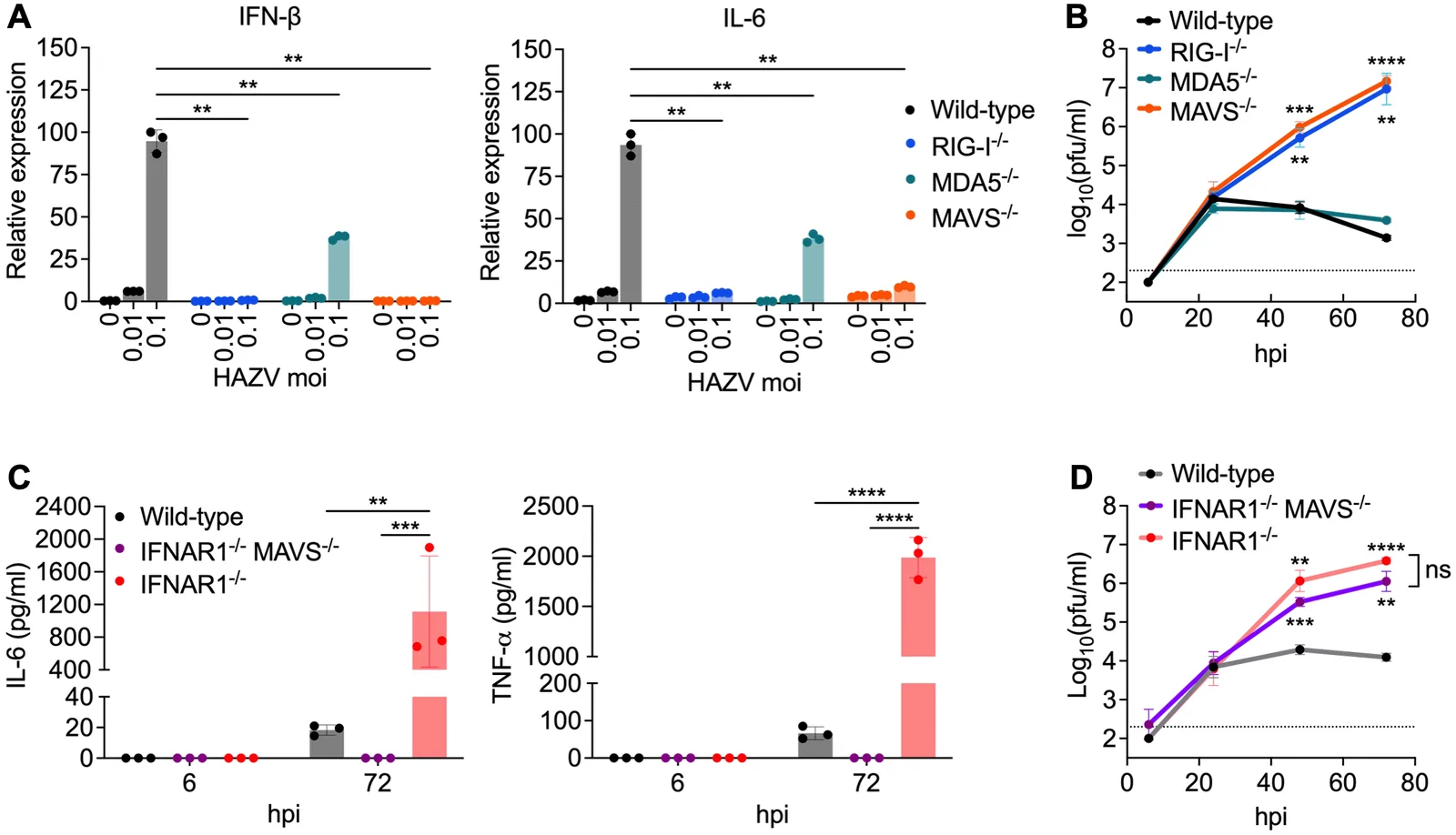
RIG-I/MAVS signaling mediates inflammatory responses during HAZV infection. **(A)** qRT-PCR analysis for the relative quantity of IFN-β and IL-6 mRNA in A549 wild-type, RIG-I^-/-^, MDA5^-/-^, and MAVS^-/-^ cells infected with HAZV at the indicated multiplicity of infection (moi) for 24 hours. **(B)** Virus loads of HAZV in the culture media from A549 wild-type, RIG-I^-/-^, MDA5^-/-^ and MAVS^-/-^ cells at the indicated time points. The detection limit is indicated as a dotted line. **(C)** IL-6 and TNF-α levels in the culture media from HAZV-infected wild-type, IFNAR1^-/-^, and IFNAR1^-/-^ MAVS^-/-^ mouse bone marrow-derived macrophages at the indicated time points. **(D)** Virus loads of HAZV in the culture media from wild-type, IFNAR1^-/-^, and IFNAR1^-/-^ MAVS^-/-^ mouse bone marrow-derived macrophages at the indicated time points. The detection limit is indicated as a dotted line. (A-D) Data are shown as mean ± SD of biological triplicate experiments. The *p*-values were calculated using a two-way ANOVA with Tukey’s multiple comparisons test. ns, not significant, **p < 0.01, ***p < 0.001, ****p < 0.0001.

HAZV infection model utilizing IFNAR1^-/-^ mice demonstrated that inflammatory cytokine levels correlated with the liver damage (Fig 1E–1G). To clarify the contribution of the inflammatory immune response to the liver damage development, we first attempted to determine the signaling pathways involved in cytokine production during HAZV infection in cell cultures. Cytoplasmic RNA sensing molecules, including retinoic acid-inducible gene-I (RIG-I) and melanoma differentiation-associated protein 5 (MDA5), play a critical role in inducing cytokines in cells infected with RNA viruses [26–28]. These two molecules use the same adaptor protein, mitochondrial antiviral signaling protein (MAVS), to transmit the signal to the downstream cascade [29–32]. We therefore measured cytokine expression in human lung carcinoma A549 cell lines that are deficient in RIG-I, MDA5, or MAVS, as well as parental A549 cell line (wild-type), following HAZV infection (Fig 2A). IFN-β and IL-6 mRNA induction was drastically reduced by RIG-I or MAVS knockout, while partial reduction of these cytokines was observed with MDA5 knockout (Fig 2A). Virus loads in the culture medium of RIG-I or MAVS knockout cells were significantly higher than in wild-type or MDA5 knockout cells (Fig 2B). We further measured IL-6 and TNF-α in the culture medium of mouse bone marrow-derived macrophages (BMDM) following HAZV infection (Fig 2C). IFNAR1^-/-^ BMDM secreted higher amounts of these cytokines than wild-type BMDM. In line with the results obtained with A549 cell lines, in MAVS deficient IFNAR1^-/-^ BMDM (IFNAR1^-/-^ MAVS^-/-^ BMDM), the cytokine production was significantly reduced. Virus loads in the culture medium of IFNAR1^-/-^ BMDM were higher than that of wild-type BMDM, but it was comparable to that of IFNAR1^-/-^ MAVS^-/-^ BMDM (Fig 2D). Collectively, RIG-I/MAVS signaling primarily contributes to the inflammatory immune response during HAZV infection.

### A deficiency in the MAVS-dependent inflammatory cytokine response cannot eliminate liver damage following HAZV infection

**Fig 3:**
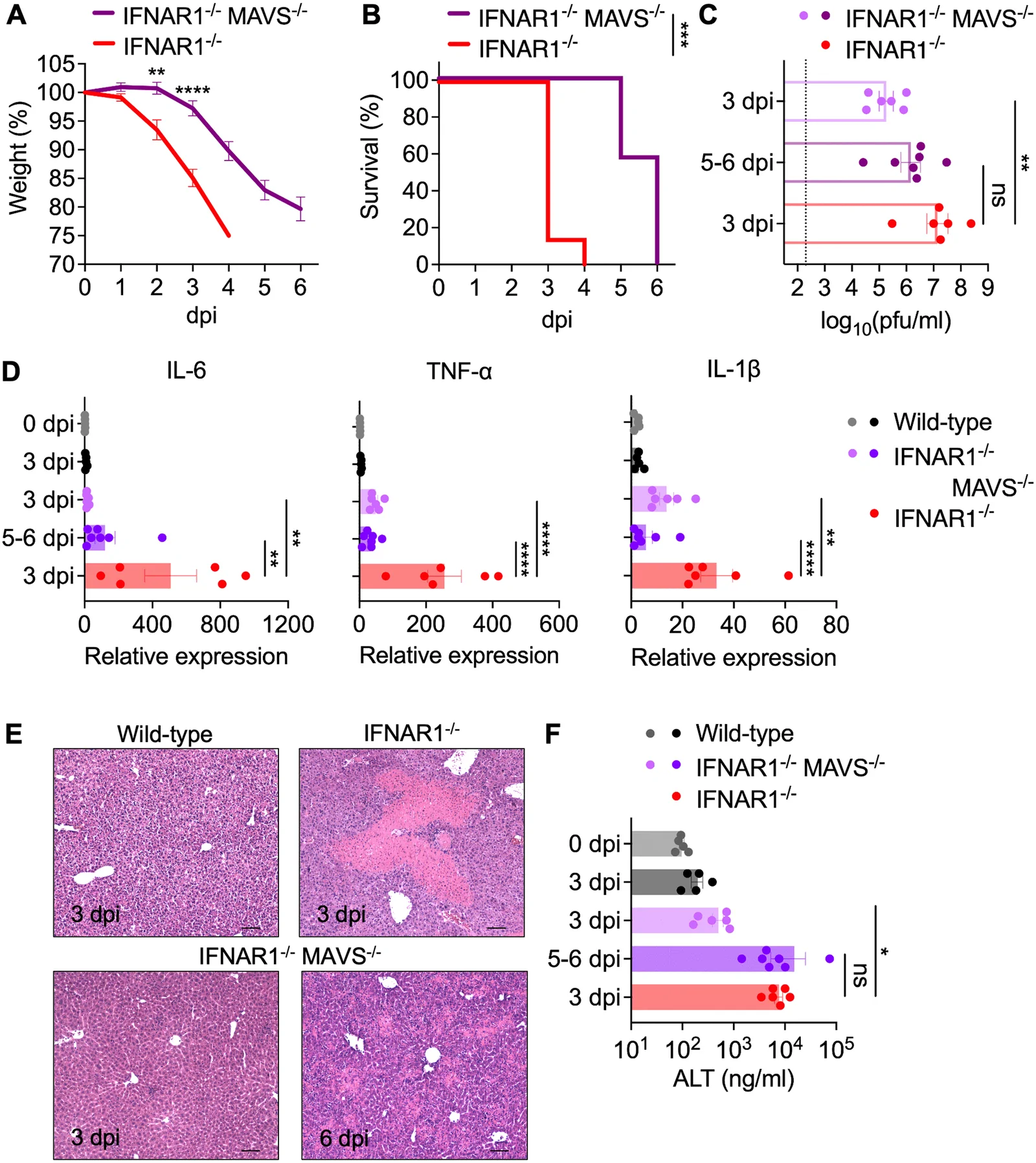
IFNAR1^-/-^ mice lacking MAVS signaling still develop liver damage and weight loss following HAZV infection. **(A, B)** Weight change (A) or survival (B) of IFNAR1^-/-^ mice and IFNAR1^-/-^ MAVS^-/-^ mice following HAZV infection (n = 7/each group). **(C)** Virus loads of HAZV in the blood from IFNAR1^-/-^ MAVS^-/-^ mice (n = 6 at 3 dpi, n = 7 at 5–6 dpi) and IFNAR1^-/-^ mice (n = 6) at the indicated time points. The detection limit is indicated as a dotted line. **(D)** qRT-PCR analysis for the relative quantity of IL-6, TNF-α, and IL-1β mRNA in the livers of uninfected wild-type mice (n = 5), and of HAZV-infected wild-type (n = 5), IFNAR1^-/-^ MAVS^-/-^ mice (n = 6 at 3 dpi, n = 7 at 5–6 dpi) and IFNAR1^-/-^ mice (n = 6) at the indicated time points. **(E)** Representative H&E staining images of liver sections from HAZV-infected wild-type, IFNAR1^-/-^ and IFNAR1^-/-^ MAVS^-/-^ mice at the indicated time points. Scale Bar: 100 μm. **(F)** ALT levels in the plasma from uninfected wild-type mice (n = 5) or HAZV-infected wild-type (n = 5), IFNAR1^-/-^ MAVS^-/-^ mice (n = 6 at 3 dpi, n = 7 at 5–6 dpi) and IFNAR1^-/-^ (n = 6) mice at the indicated time points. (A-D, and F) Data are pooled from two independent experiments and shown as mean ± SEM. The *p*-values were calculated using a two-way ANOVA with Tukey’s multiple comparisons test (A), log-rank test (B), one-way ANOVA with Tukey’s multiple comparison test (C, D), Welch’s ANOVA with Dunnett’s T3 multiple comparison test (F). ns, not significant, *p < 0.05, **p < 0.01, ***p < 0.001, ****p < 0.0001.

An uncontrolled inflammatory immune response, known as ‘cytokine storm’, can lead to systemic or local organ damage and dysfunction. It is currently thought that cytokine storm is the main factor leading to liver damage and lethal outcomes during CCHF. Since our *in vitro* experiments revealed that RIG-I/MAVS signaling is a major source of inflammatory cytokines during HAZV infection, we compared disease progression in IFNAR1^-/-^ mice and IFNAR1^-/-^ MAVS^-/-^ mice following HAZV infection. Notably, the initiation of lethal outcomes, including weight loss and lethality, was clearly delayed by MAVS deficiency: IFNAR1^-/-^ mice showed over 20% of weight loss at 3–4 dpi; in contrast, IFNAR1^-/-^ MAVS^-/-^ mice showed it at 5–6 dpi (Fig 3A, 3B). Blood virus loads in IFNAR1^-/-^ MAVS^-/-^ mice at 3 dpi were significantly lower than that IFNAR1^-/-^ mice at 3 dpi, but the difference was not significant at 5–6 dpi (Fig 3C). Consistent with the *in vitro* results, the expression of inflammatory cytokines such as IL-6, TNF-α, and IL-1β in the livers of IFNAR1^-/-^ MAVS^-/-^ mice at 3 and 5–6 dpi was significantly lower than in IFNAR1^-/-^ mice at 3 dpi (Fig 3D). Histopathological analysis revealed that IFNAR1^-/-^ MAVS^-/-^ mice at 3 dpi did not exhibit apparent liver pathology, with only small foci of infiltrating immune cells observed, similar to wild-type mice (Fig 3E). In contrast, extensive hepatocellular necrosis was evident in IFNAR1^-/-^ MAVS^-/-^ mice at 6 dpi, comparable to IFNAR1^-/-^ mice at 3 dpi (Fig 3E). Plasma ALT levels in IFNAR1^-/-^ MAVS^-/-^ mice increased drastically from 3 dpi to 5–6 dpi and eventually reached the same levels as in IFNAR1^-/-^ mice at 3 dpi (Fig 3F). Collectively, these findings indicate that MAVS-dependent cytokine storm partially contributes to liver damage during HAZV infection, suggesting that additional pathogenic factors are also involved.

### Virus replication directly mediates Kupffer cell loss associated with liver damage in IFNAR1^-/-^ mice

**Fig 4:**
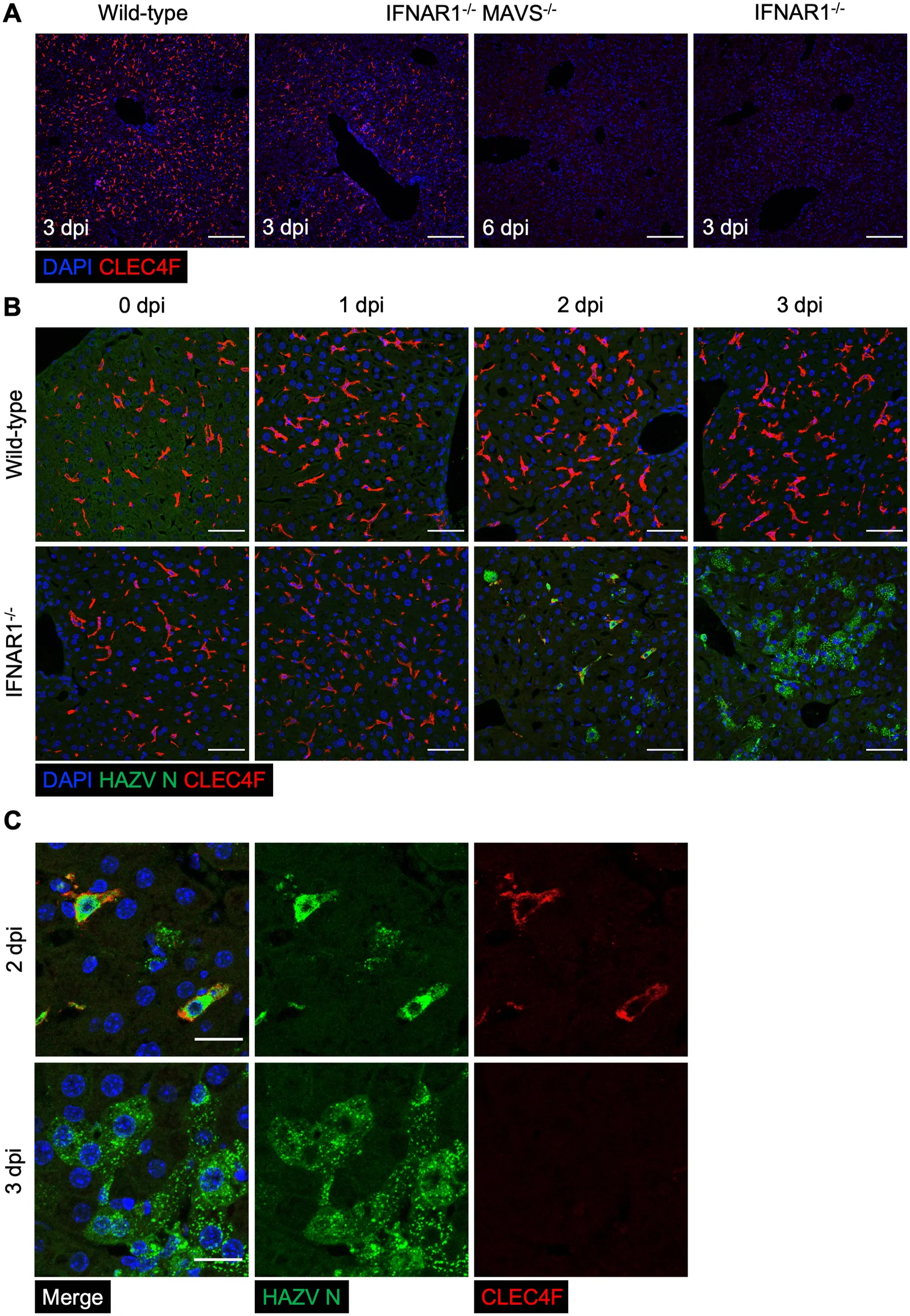
HAZV replication triggers Kupffer cell loss following HAZV infection. **(A)** Representative immunostaining images of liver sections from HAZV-infected wild-type, IFNAR1^-/-^ and IFNAR1^-/-^ MAVS^-/-^ mice at the indicated time points. Scale bar: 200 μm. **(B)** Representative immunostaining images of liver sections from wild-type and IFNAR1^-/-^ mice at the indicated time points. Scale bars: 50 μm. **(C)** Enlarged images of liver sections from IFNAR1^-/-^ mice shown in (B). Scale bars: 20 μm.

To identify a new factor involved in liver damage caused by HAZV infection, we performed an immunofluorescence analysis of HAZV-infected liver (Fig 4A). In CCHF patients, mononuclear phagocytes represent one of the antigen-positive cell populations; thus, we investigated the presence of Kupffer cell population in the liver of HAZV-infected mice. Interestingly, in the liver of IFNAR1^-/-^ mice, CLEC4F-positive Kupffer cells mostly disappeared at 3 dpi, in contrast to wild-type mice (Fig 4A). Reduction of the Kupffer cell population was also observed in the liver of IFNAR1^-/-^ MAVS^-/-^ mice, although its initiation was significantly delayed compared to IFNAR1^-/-^ mice (Fig 4A). Gene expression of CLEC4F mRNA in the livers also indicated a reduction in the number of Kupffer cells in IFNAR1^-/-^ mice and IFNAR1^-/-^ MAVS^-/-^ mice (S1A Fig). Notably, the kinetics of Kupffer cell population correlated with the severity of liver damage (Figs 3E, 3F, and 4A). Thus, we hypothesized that, in addition to cytokine storm-mediated cell death, cytokine storm-independent Kupffer cell loss might be responsible for fatal liver damage. To investigate the mechanism by which HAZV infection causes Kupffer cell loss, we used immunofluorescence analysis to determine which cell types are infected with HAZV in the liver (Fig 4B and 4C). Liver samples from wild-type or IFNAR1^-/-^ mice at the indicated time points were stained with anti-HAZV nucleocapsid protein (N) antibody and anti-CLEC4F antibody. HAZV antigen-positive cells were not detected in the wild-type liver, and the Kupffer cell population did not change throughout the tested time points (Fig 4B). In contrast, HAZV N was detected in Kupffer cells in IFNAR1^-/-^ mice at 2 dpi, and a clear reduction in Kupffer cell number was observed from 2 dpi to 3 dpi (Fig 4B and 4C). Interestingly, most of the HAZV N-positive cells were hepatocytes rather than Kupffer cells at 3 dpi. Consistent with the immunofluorescence analysis results, the expression levels of Kupffer cell-related genes CLEC4F and CD163 were significantly lower in the livers of IFNAR1^-/-^ mice from 2 dpi onwards. There was no obvious reduction in the expression of these genes in the livers of wild-type mice (S1B Fig). These results demonstrate that virus replication together with inflammatory cytokines triggers Kupffer cell loss, potentially leading to liver damage during HAZV infection.

### A deficiency of IFNAR1 in mononuclear phagocytes, but not in hepatocytes, enables the development of lethal disease associated with liver damage following HAZV infection

**Fig 5:**
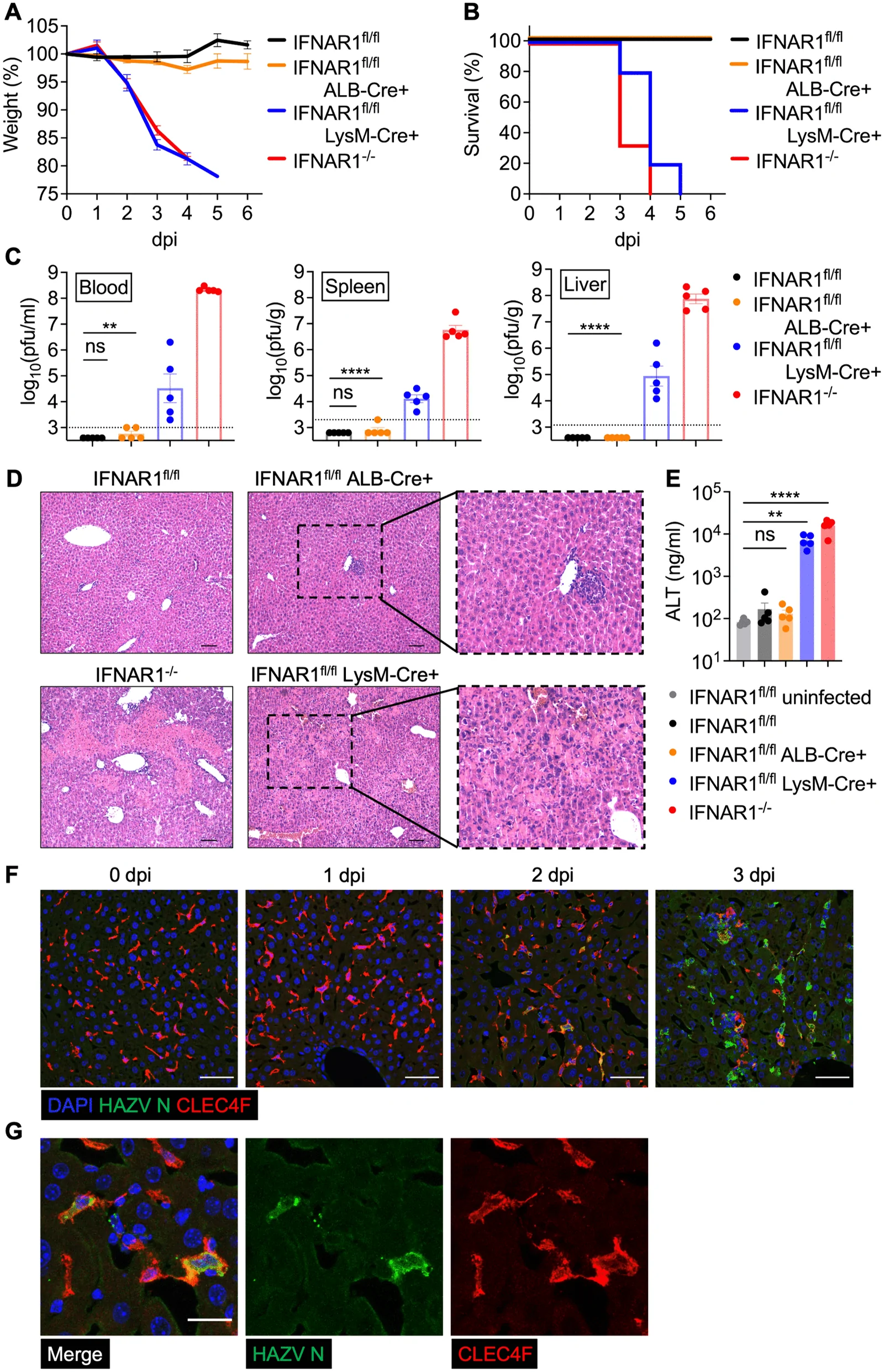
IFNAR1 deficiency in Kupffer cells, but not hepatocytes, causes fatal liver damage following HAZV infection. **(A, B)** Weight change (A) or survival (B) of IFNAR1^fl/fl^ (n = 5), IFNAR1^fl/fl^ ALB-Cre+ (n = 5), IFNAR1^fl/fl^ LysM-Cre+ (n = 5), and IFNAR1^-/-^ mice (n = 6) following HAZV infection. **(C)** Virus loads of HAZV in the blood, livers and spleens from IFNAR1^fl/fl^, IFNAR1^fl/fl^ ALB-Cre+, IFNAR1^fl/fl^ LysM-Cre+ and IFNAR1^-/-^ mice at 3 dpi (n = 5/each group). The detection limit is indicated as a dotted line. **(D)** Representative H&E staining images of the livers from HAZV-infected IFNAR1^fl/fl^, IFNAR1^fl/fl^ ALB-Cre+, IFNAR1^fl/fl^ LysM-Cre+ and IFNAR1^-/-^ mice at 3 dpi. Scale bars, 100 μm. **(E)** ALT levels in the plasma of uninfected IFNAR1^fl/fl^ or HAZV-infected IFNAR1^fl/fl^, IFNAR1^fl/fl^ ALB-Cre+, IFNAR1^fl/fl^ LysM-Cre+ and IFNAR1^-/-^ mice at 3 dpi (n = 5/each group). **(F)** Representative immunostaining images of liver sections from uninfected or HAZV-infected IFNAR1^fl/fl^ LysM-Cre+ mice at the indicated time points. Scale bars: 50 μm. **(G)** Enlarged images of liver sections from HAZV-infected IFNAR1^fl/fl^ LysM-Cre+ mice at 2 dpi shown in (F). Scale bar: 20 μm. (A, C, and E) Data are shown as mean ± SEM. The *p*-values were calculated using a one-way ANOVA with Tukey’s multiple comparison test. ns, not significant, **p < 0.01, ****p < 0.0001.

To further investigate the contribution of specific cell types to liver damage initiation during HAZV infection, we tested mice lacking IFNAR1 in hepatocytes (IFNAR1^fl/fl^ ALB-Cre+) and in the myeloid cell lineage, including monocytes, macrophages, and granulocytes (IFNAR1^fl/fl^ LysM-Cre+). To confirm the efficacy of Cre recombinase activity in the liver of LysM-Cre+ mice, we intercrossed LysM-Cre+ mice with reporter mice harboring a red fluorescent protein variant (tdTomato) gene under a loxP-flanked STOP cassette. We confirmed that CLEC4F-positive Kupffer cells mostly expressed tdTomato in the liver (S2 Fig). This result indicates that Cre recombinase in LysM-Cre+ mice is active in Kupffer cells and sufficiently deletes the target gene. Following HAZV infection, IFNAR1^fl/fl^ LysM-Cre+ mice exhibited weight loss and lethality, similar to that observed in systemic IFNAR1^-/-^ mice (Fig 5A, 5B). However, IFNAR1^fl/fl^ mice that did not carry the Cre recombinase gene, as well as IFNAR1^fl/fl^ ALB-Cre+ mice, showed no clinical symptoms, including weight loss and lethality (Fig 5A, 5B). Virus loads significantly increased in the blood, spleens, and livers of IFNAR1^fl/fl^ LysM-Cre+ mice and systemic IFNAR1^-/-^ mice, but not in IFNAR1^fl/fl^ mice or IFNAR1^fl/fl^ ALB-Cre+ mice (Figs 5C and S3A). In addition, the expression levels of IL-6, TNF-α and IL-1β in the livers of IFNAR1^fl/fl^ LysM-Cre+ mice, but not in IFNAR1^fl/fl^ ALB-Cre+ mice, were clearly increased at 3 dpi, consistent with observations in IFNAR1^-/-^ mice (S3B Fig). Histopathological analysis of the liver at 3 dpi revealed hepatocellular necrosis in IFNAR1^fl/fl^ LysM-Cre+ mice, similar to that observed in systemic IFNAR1^-/-^ mice (Fig 5D). In contrast, IFNAR1^fl/fl^ mice and IFNAR1^fl/fl^ ALB-Cre+ mice showed no obvious histopathological abnormality, although small foci of infiltrating immune cells were occasionally present (Fig 5D). Consistent with the histopathological observation, increased ALT levels were observed in IFNAR1^fl/fl^ LysM-Cre+ mice and systemic IFNAR1^-/-^ mice, but not in the other mouse lines (Fig 5E).

Immunofluorescence analysis further revealed that HAZV N was found in CLEC4F-positive Kupffer cells at 2 dpi, followed by a marked reduction in Kupffer cells at 3 dpi in the liver of IFNAR1^fl/fl^ LysM-Cre+ mice (Fig 5F, 5G). Consistently, CLEC4F and CD163 mRNA levels were significantly decreased in the liver of IFNAR1^fl/fl^ LysM-Cre+ mice as observed in systemic IFNAR1^-/-^ mice (S3C Fig). In contrast, there was no reduction in these gene levels in the livers of IFNAR1^fl/fl^ and IFNAR1^fl/fl^ ALB-Cre+ mice at 3 dpi. To further confirm that HAZV infection in Kupffer cells triggers lethal outcomes, we tested IFNAR1^fl/fl^ CX3CR1-Cre+ mice predominantly lacking IFNAR1 in monocytes and tissue resident macrophages including Kupffer cells with HAZV infection [33]. Similar to IFNAR1^fl/fl^ LysM-Cre+ mice, IFNAR1^fl/fl^ CX3CR1-Cre+ mice exhibited lethal outcomes including weight loss, lethality (S4A, S4B Fig), and high viral loads in the blood (S4C Fig). These results demonstrate that IFNAR1 signaling deficiency in Kupffer cells results in virus spread and liver damage during HAZV infection.

### Kupffer cell loss is a potential driver of virus spread to hepatocytes and fatal liver damage during CCHFV infection

**Fig 6:**
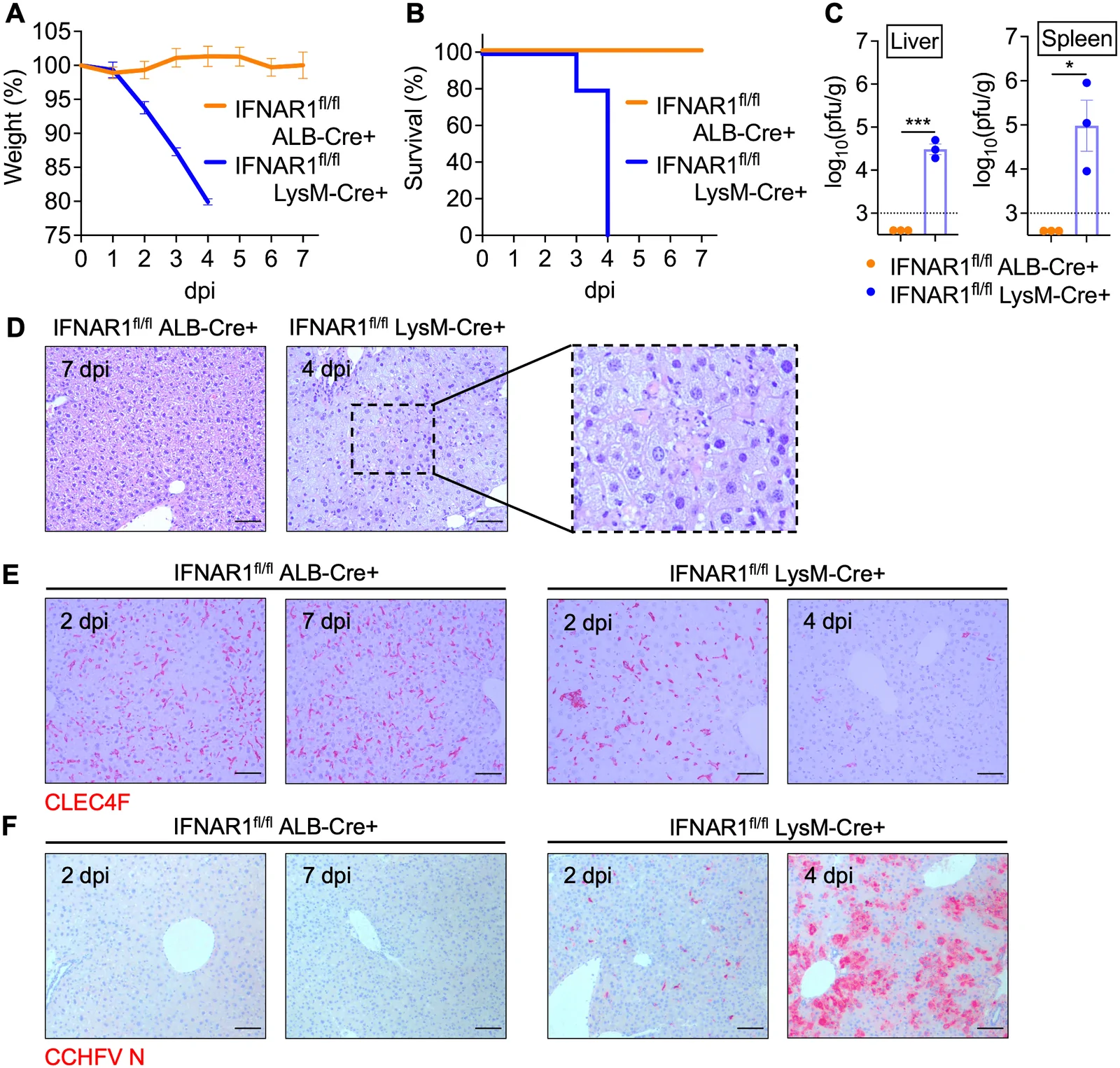
IFNAR1^fl/fl^ LysM-Cre+ mice develop lethal disease following CCHFV infection. **(A, B)** Weight change (A) or survival (B) of IFNAR1^fl/fl^ ALB-Cre+ (n = 6) and IFNAR1^fl/fl^ LysM-Cre+ (n = 5) following CCHFV infection. **(C)** Virus loads of CCHFV in the livers and spleens from IFNAR1^fl/fl^ ALB-Cre+ (n = 3) and IFNAR1^fl/fl^ LysM-Cre+ mice (n = 3) at 2 dpi. The detection limit is indicated as a dotted line. **(D)** Representative H&E staining images of the livers from CCHFV-infected IFNAR1^fl/fl^ ALB-Cre+ and IFNAR1^fl/fl^ LysM-Cre+ mice at 7 and 4 dpi, respectively. Scale bars: 100 μm. **(E, F)** Representative immunostaining images oflivers from CCHFV-infected IFNAR1^fl/fl^ ALB-Cre+ and IFNAR1^fl/fl^ LysM-Cre+ mice. Scale bars: 100 μm. (A, C) Data are shown as mean ± SEM. The *p*-values were calculated using a one-way ANOVA with Tukey’s multiple comparison test. *p < 0.05, ***p < 0.001.

To clarify the contribution of Kupffer cells to viral spread and damage in the liver during CCHFV infection, we also tested IFNAR1^fl/fl^ ALB-Cre+ mice and IFNAR1^fl/fl^ LysM-Cre+ mice with authentic CCHFV. As with HAZV infection cases, IFNAR1^fl/fl^ LysM-Cre+ mice exhibited weight loss and lethality; in contrast, IFNAR1^fl/fl^ ALB-Cre+ mice exhibited no clinical symptoms (Fig 6A, 6B). High viral loads were detected in the livers and spleens of IFNAR1^fl/fl^ LysM-Cre+ mice, but not in those of IFNAR1^fl/fl^ ALB-Cre+ mice (Fig 6C). Histopathological analysis revealed no pathological abnormality in the liver of IFNAR1^fl/fl^ ALB-Cre+ mice at 7 dpi. However, hepatocellular necrosis was observed in IFNAR1^fl/fl^ LysM-Cre+ mice at 4 dpi (Fig 6D). CCHFV infection also induced a reduction in CLEC4F-positive Kupffer cells in the livers of IFNAR1^fl/fl^ LysM-Cre+ mice, but not in those of IFNAR1^fl/fl^ ALB-Cre+ mice (Fig 6E). In addition, CCHFV antigens were predominantly detected in morphologically identified Kupffer cells at 2 dpi and then spread to hepatocytes at 4 dpi, when Kupffer cells were absent, in the livers of IFNAR1^fl/fl^ LysM-Cre+ mice (Fig 6F). These results strongly suggest that Kupffer cells play a central role in controlling viral spread in the liver, and that the loss of Kupffer cells is a key determinant of liver damage driven by viral spread to hepatocytes during CCHFV infection.

## Discussion

CCHF is a hemorrhagic disease of worldwide concern with a high fatality rate; however, the pathogenesis of CCHF has not been fully understood. To address it, in this study, we sought to clarify the fundamental mechanism underlying fatal liver damage during CCHF using immunodeficient mice. Particularly, in addition to widely used systemic IFNAR1^-/-^ mice, we employed cell specific IFNAR1^-/-^ mice to more precisely examine the contribution of individual CCHFV-targeted cell type to liver damage. Throughout analysis of both systemic and selective IFNAR1^-/-^ mouse models following HAZV and CCHFV infection, we elucidated that type I interferon signaling in mononuclear phagocytes including Kupffer cells is critical for preventing virus spread in the liver and raised the possibility that inflammatory cytokine- and virus replication-mediated Kupffer cell loss results in viral spread to hepatocytes and subsequent fatal liver damage during CCHFV infection.

Kupffer cells are resident macrophages in the liver and represent approximately 35% of nonparenchymal liver cells in adult mice [34]. Kupffer cells adhere to the liver sinusoidal endothelial cells (LSECs), capturing pathogens and dead cells from the blood stream. Previous studies demonstrate that several viruses, including lymphocytic choriomeningitis virus (LCMV) and adenovirus, can be rapidly captured by Kupffer cells [35–37]. Interestingly, in the case of LCMV infection, transient depletion of Kupffer cells (e.g., via administration of clodronate liposome) disrupts viral clearance, leading to viral spread from Kupffer cells to hepatocytes [35]. In addition, it has been reported that Kupffer cell depletion by clodronate liposome inoculation reduces the viability of LSECs in mice with sepsis [38]. Taken together, these previous studies suggest that the loss of Kupffer cells result in increased virus loads in the bloodstream and enhances the opportunities for virus invasion of hepatocytes by compromising the integrity of LSECs during virus infections. In this study, we found that IFNAR1 deficiency in mononuclear phagocytes including Kupffer cells, but not hepatocytes, leads Kupffer cell loss associated with liver damage and lethal disease during HAZV and CCHFV infection, even though both viruses have the potential to infect hepatocytes in mouse models. Importantly, in CCHFV-infected IFNAR1^fl/fl^ LysM-Cre+ mice, virus antigens were initially detected in Kupffer cells and subsequently spread to hepatocytes, those with intact IFNAR1 signaling, when Kupffer cells disappeared as observed in systemic IFNAR1^-/-^ mice following HAZV infection (Figs 4B and 6F). Our findings strongly suggest that Kupffer cells act as gatekeepers to protect hepatocytes against virus invasion, and that the loss of Kupffer cells driven by host inflammatory responses and virus replication cells play an initiating role in fatal liver damage during CCHFV infection.

In this study, we used selective IFNAR1^-/-^ mice to delineate the contribution of Kupffer cells and hepatocytes to liver damage driven by CCHFV infection, using IFNAR1^fl/fl^ LysM-Cre+ mice and IFNAR1^fl/fl^ CX3CR1-Cre+ mice for Kupffer cells and IFNAR1^fl/fl^ ALB-Cre+ mice for hepatocytes. These Cre recombinase-expressing mouse lines are widely used to investigate the effects of a target gene in specific cell types, and the specificity of Cre recombinase expression has been well characterized [33,39–43]. Although adult ALB-Cre+ mice specifically express Cre recombinase in hepatocytes, LysM-Cre+ mice and CX3CR1-Cre+ mice exhibit broader its expression across mononuclear phagocytes including monocytes and macrophages. The previous study shows that recruited monocytes/macrophages are an initial target of CCHFV infection and that monocytes recruitment-related chemokines such as C-C motif ligand (CCL) 2, CCL3, and CCL5 are significantly elevated with the disease severity in a mouse model [44]. These findings suggest that recruited monocytes may also have pathogenic effects in the liver during CCHFV infection. Given the wide range of Cre recombinase expression in LysM-Cre+ mice and CX3CR1-Cre+ mice, we cannot clarify this point in this study; however, we speculate that recruited monocytes/macrophages in the liver facilitate Kupffer cell loss and liver damage by producing viral progeny and inflammatory cytokines. Further studies are needed to determine whether inhibition of monocyte recruitment by treatment with antagonists against those chemokines or their receptors ameliorates liver pathology in the mouse model. Interestingly, in addition to monocytes, the numbers of CD8+ T cells and natural killer cells are significantly elevated in the livers of CCHFV-infected mice at the late phase of infection. These cell types are cytotoxic immune cells that play critical roles in antiviral immunity. However, under conditions of widespread viral infection, excessive activation of these cells would result in severe tissue damage or organ dysfunction by inducing extensive cell death. Therefore, further studies investigate the roles of these immune cells to fully understand the mechanisms underlying liver damage during CCHFV infection.

This study has not unveiled the detailed molecular mechanism of Kupffer cell loss. IFNAR1^-/-^ MAVS^-/-^ mice, which lack the ability to produce cytokines, still exhibited Kupffer cell loss following HAZV infection, but the process was clearly delayed compared to IFNAR1^-/-^ mice (Fig 4A). Based on this result, we propose that loss of Kupffer cell is caused by two distinct mechanisms: 1) inflammatory cytokine-mediated cell death (Fig 4A) and 2) virus replication-mediated cell death (Figs 5F, 5G, 6F). Clinical findings indicate that CCHF patients exhibit increased levels of inflammatory cytokines associated with disease severity. Particularly, serum levels of IL-6, IL-10, IFN-γ and TNF-α are significantly higher in patients with severe disease than in patients with moderate disease or survivors [12,45,46]. TNF-α is a well-known cytokine involved in cell death under inflammatory conditions [47]. A previous study showed that deletion of TNF-α receptor genes (TNFAR1/2) or treatment with a neutralizing antibody against TNF-α partially increased viability of Kupffer cells and improved the survival of IFNAR1-blocked mice following CCHFV infection [23]. These results strongly suggest that TNF-α is a potent inducer of cell death for Kupffer cells during CCHFV infection. Interestingly, as there are several reports of TNF-α and IFN-γ, among various inflammatory cytokines, synergistically inducing cell death *in vitro* and *in vivo* [48,49], it is speculated that multiple cytokines must be targeted to prevent the loss of Kupffer cells during CCHFV infection. In addition to the contribution of inflammatory cytokines to Kupffer cell loss, we propose that CCHFV replication itself is also its causative factor. Several human cell lines and primary cell types, such as monocyte-derived dendritic cells and macrophages, are susceptible to varying degrees *in vitro* [50–52]. In particular, the human adrenal gland/cortex carcinoma SW-13 cell line, which is generally used for CCHFV propagation, and the human hepatocarcinoma Huh-7 cell line exhibit a cytopathic effect following CCHFV infection [50,53,54]. *In vitro*, it has been suggested that CCHFV can induce apoptosis through ER stress, such as the unfolded protein response, in Huh-7 cells [54]. In summary, the loss of Kupffer cell during CCHFV infection may result from the activation of intrinsic and extrinsic cell death pathways.

A previous study reveals that MAVS deficient mice treated with anti-IFNAR1 blocking antibody show significant weight loss but do not succumb to CCHFV infection [23]. In contrast, in our study, all IFNAR1^-/-^ MAVS^-/-^ mice reached the humane endpoint criteria and were euthanized following HAZV infection (Fig 3A, 3B). The basis for this discrepancy remains unclear; however, we speculate that differences in the experimental setting may account for it. Genetic IFNAR1 deletion, compared with antibody-mediated IFNAR1-blockage, may more effectively abrogate type I interferon-dependent antiviral immunity and result in increased susceptibility to virus infection. In addition, differences of inoculated virus dose may also contribute. Despite this discrepancy, both our study and the previous study suggest that MAVS-dependent inflammatory responses play a key role in the development of fatal liver damage and CCHF pathogenesis. To accelerate the development of inflammatory cytokine-targeted therapeutics for CCHF, further studies are needed to define the individual contributions of inflammatory cytokines to disease pathogenesis.

CCHF is one of the important life-threatening infectious diseases in humans, and there is an urgent need to discover specific therapeutics for CCHF. In this study, we propose that MAVS-controlled cytokine storm and virus replication synergistically contribute to the loss of Kupffer cells and viral spread to hepatocytes, leading to fatal acute liver damage during CCHFV infection. Further investigations are required to evaluate the efficacy of anti-TNF-α neutralizing treatment, either alone or in combination with small-molecule inhibitors of intrinsic cell death pathway and/or antiviral drugs such as ribavirin. Such studies could help to advance the development of therapeutics targeting liver damage against CCHF. Of note, our findings in this study are largely based on experiments using immunodeficient mouse models, and there might be discrepancies between observations in humans and those in these models. For instance, biopsy studies demonstrate that CCHFV antigens are founded in Kupffer cells in addition to hepatocytes and LSEC, which suggesting that Kupffer cells still exist in fatal cases in contrast to findings in lethal mouse models of CCHF. This discrepancy may be due to the different degree of the loss of Kupffer cells between humans and mouse models and/or the virus strain. Given the limited number of biopsy studies, further analyses or comparisons using human-derived materials or physiologically relevant animal models such as immunocompromised mice or nonhuman primates will be required to deeply understand liver pathology driven by CCHFV infection.

## Materials and methods

### Ethics statement

All animal experiment protocols and procedures were approved by the Institutional Animal Care and Use Committee at both institutions. Approved animal protocol number: 81-02.04.2019-A247 for HAZV experiments (University Hospital Bonn) and 1906055B for CCHFV experiments (The University of Texas Medical Branch at Galveston).

### Cells and viruses

Human adrenal gland/cortex carcinoma SW-13 cells and human alveolar adenocarcinoma A549 wild-type, RIG-I^-/-^, MDA5^-/-^, and MAVS^-/-^ cells (InvivoGen, a549d-nfis, a549d-korigi, a549d-komda5, and a549d-komavs, respectively) were maintained in Dulbecco’s modified Eagle’s medium (DMEM) (Thermo Fisher Scientific, 41965062) supplemented with 10% heat-inactivated fetal bovine serum (FBS) (Thermo Fisher Scientific, 1027010), 100 units/ml penicillin, and 100 μg/ml streptomycin (Thermo Fisher Scientific, 15140122) at 37 °C and 5% CO_2_. Hazara virus (HAZV) strain JC280 and Crimean-Congo hemorrhagic fever virus (CCHFV) strain IbAr10200 provided by Dr. Thomas G. Ksiazek at The University of Texas Medical Branch at Galveston, Texas, United States were propagated in SW-13 cells.

### Mice

Wild-type C57BL/6 mice were purchased from Charles River Laboratories, and IFNAR1^fl/fl^, LysM-Cre+, ALB-Cre+, CX3CR1-Cre+, and ROSA26-tdTomato mice were purchased from The Jackson Laboratory. IFNAR1^-/-^ mice and MAVS^-/-^ mice were provided by Dr. Takashi Fujita, Kyoto University, Kyoto, Japan. To obtain IFNAR1^-/-^ MAVS^-/-^ mice, IFNAR1^-/-^ mice were intercrossed with MAVS^-/-^ mice. To obtain cell type-specific IFNAR1-deficient mice, IFNAR1^fl/fl^ mice were intercrossed with the above Cre+ mouse lines. All mice were housed in a specific-pathogen free mouse facility at University Hospital Bonn and The University of Texas Medical Branch at Galveston according to institutional and governmental animal welfare guidelines.

### *In vivo* virus infection

Mice were intraperitoneally inoculated with 1000 plaque forming unit (PFU) of HAZV or subcutaneously inoculated with 1000 PFU of CCHFV. After inoculation, weight and temperature of the mice were monitored every day. The animal experiments using HAZV or CCHFV were performed in a biosafety level 3 facility at University Hospital Bonn or in a biosafety level 4 facility at The University of Texas Medical Branch at Galveston, respectively.

### Preparation of mouse bone marrow-derived macrophages

Bone marrow cells isolated from tibias and femurs were treated with ACK Lysis Buffer (Thermo Fisher Scientific, A1049201) to remove red blood cells. The cells were washed and resuspended with DMEM supplemented with 10% FBS, 100 units/ml penicillin, and 100 μg/ml streptomycin, and 50 ng/ml mouse macrophage colony-stimulating factor (M-CSF) (R&D Systems, 416-ML). Half of the culture medium was replaced with fresh medium containing 10% FBS, 100 units/ml penicillin, and 100 μg/ml streptomycin, and 100 ng/ml M-CSF at day 4 of culture. The cells were harvested for the experiments at day 8 of culture.

### Virus titration

Cell culture supernatants, supernatants of mouse tissue homogenates, and mouse whole blood collected into blood collection tubes containing K3-EDTA (Sarstedt, 41.1504.005) or serum from mouse blood collected into BD Microtainer SST tubes (BD, 365967) were used for the virus titration. For mouse tissue homogenates, HAZV-infected and CCHFV-infected mouse tissues were homogenized in DMEM supplemented with 2% FBS and 100 units/ml penicillin, and 100 μg/ml streptomycin using Tissue Grinder Mixy Professional (NIPPON Genetics, NG010) and using TissueLyser II (QIAGEN, 85300), respectively. After removing cell debris by centrifugation, the homogenate supernatants were subjected to the virus titration. HAZV and CCHFV titers were determined by standard plaque assay using SW-13 cells. Serially diluted HAZV with DMEM containing 2% FBS and 100 units/ml penicillin, and 100 μg/ml streptomycin was incubated with the cells for 1 hour at 37°C. After washing out the inoculum, the cells were overlaid with DMEM containing 2% FBS, 100 units/ml penicillin, and 100 μg/ml streptomycin, and 1.5% Avicel PH-101 (Sigma, 11365). At 4 days post infection, the cells were fixed with 6% formaldehyde in PBS and stained with crystal violet. For CCHFV titration, the cells were incubated with serially diluted CCHFV for 30 minutes at 37°C, then washed and overlaid with minimal essential medium (MEM) containing 5% FBS, 100 units/ml penicillin, and 100 μg/ml streptomycin, and 1% Avicel RC-591 NF (DuPont, RC591-NFBA500). At 3 days post infection, the cells were fixed with 10% neutral buffered formalin and stained with crystal violet. The viral titers were presented as plaque forming unit (PFU).

### Quantitative real-time PCR (qRT-PCR)

Liver specimens were homogenized in TRIzol (Invitrogen, 15596) by a gentleMACS Octo Dissociator (Miltenyi Biotec, 130-096-427). Cells were suspended in TRIzol. Liver homogenates and cell suspensions were subjected to RNA extraction according to the manufacturer’s protocol. cDNA was generated using a High-Capacity cDNA Reverse Transcription Kit (Applied Biosystems, 43688). qRT-PCR was performed on a StepOnePlus Real-Time PCR System (Applied Biosystems) using TaqMan Fast Advanced Master Mix (Applied Biosystems, 4444558) or Fast SYBR Green Master Mix (Applied Biosystems, 4385614). The following TaqMan probes purchased from Thermo Fisher Scientific were used: eukaryotic 18S ribosomal RNA (Hs99999901_s1), human IFN-β (Hs01077958_s1), human IL-6 (Hs00174131_m1), mouse IL-6 (Mm01210733_m1), mouse TNF-α (Mm00443258_m1), and mouse IL-1β (Mm00434228_m1). The following primer set was used for HAZV N RNA quantification: forward primer 5’-TGGAGAAAGGATGTCGGCTT-3’, and reverse primer 5’-ATTGATCACTGTCCCTGGCA-3’. Gene expression levels were normalized to 18S ribosomal RNA levels and calculated by the ΔΔC_T_ method.

### Liver histology

HAZV-infected liver specimens were fixed with 4% paraformaldehyde in PBS and subjected to embedding in either paraffin or Tissue-Tek O.C.T. Compound (Sakura, 4583). The paraffin-embedded liver sections were stained with hematoxylin and eosin (H&E). The frozen tissue sections were rehydrated in PBS, permeabilized with 0.1% Triton X-100 in PBS, and then incubated with 10% normal donkey serum in PBS for blocking. The sections were stained with the following reagents: anti-CLEC4F antibody (R&D Systems, AF2784), anti-CCHFV N/HAZV N cross-reactive polyclonal antibody (produced in-house), Alexa Fluor 488-conjugated anti-goat IgG antibody (Jackson ImmunoResearch, 705-545-003), Alexa Fluor 568-conjugated anti-goat IgG antibody (Invitrogen, A11057), Alexa Fluor 488-conjugated anti-rabbit IgG antibody (Invitrogen, A21206), and 4′,6-diamidino-2-phenylindole (DAPI) (Thermo Fisher Scientific, D1306). CCHFV-infected liver specimens were fixed with formalin in PBS and subjected to paraffin embedding. The sections were processed either for H&E staining or for immunohistochemistry as described below. After deparaffinization, the sections were immersed in Target Retrieval Solution (DAKO, S1699) and boiled in a rice cooker for heat-induced antigen retrieval. The sections were stained with anti-CLEC4F antibody (Invitrogen, PA5–47396), anti-CCHFV N antibody (IBT Bioservices, 04–0011), alkaline phosphatase (AP)-conjugated anti-goat IgG antibody (Cell Signaling Technology, 26927), and alkaline phosphatase (AP)-conjugated anti-rabbit IgG antibody (Cell Signaling Technology, 18653) followed by incubation with AP substrate (Cell Signaling Technology, 76713). The sections were counterstained with hematoxylin. The microscopic images were acquired using a light microscope (Nikon, ECLIPSE Ci-L model) or an SP8 confocal microscope (Leica).

### Measurement of alanine aminotransferase (ALT) levels

Blood from mice was collected into blood collection tubes containing K3-EDTA, and then plasma was separated by centrifugation. The plasma was subjected to measuring ALT levels using Mouse ALT ELISA Kit (Abcam, ab282882) according to the manufacturer’s protocol.

### Enzyme-linked immunosorbent assay (ELISA)

Cell culture supernatants were cleared by centrifugation and subjected to ELISA for measuring IL-6 and TNF-α levels. The supernatants were analyzed with Mouse IL-6 DuoSet ELISA kit (R&D Systems, DY406) and Mouse TNF-alpha DuoSet ELISA kit (R&D Systems, DY410) according to the manufacturer’s protocol.

### Statistical analysis

Data were analyzed using GraphPad Prism 10 software. Statistical tests as indicated in the figure legends were performed to obtain the indicated *p*-values as follows: ns (not significant, *p* > 0.05), *p < 0.05, **p < 0.01, ***p < 0.001, ****p < 0.0001.

## Supporting information

S1 Fig HAZV infection reduces the expression of Kupffer cell marker genes. (A) qRT-PCR analysis for relative mRNA quantity of CLEC4F mRNA in the livers from uninfected wild-type mice (n = 5) or HAZV-infected wild-type (n = 5), IFNAR1^-/-^ MAVS^-/-^ mice (n = 6 at 3 dpi, n = 7 at 5–6 dpi) and IFNAR1^-/-^ mice (n = 6) at indicated time points. (B) qRT-PCR analysis for relative mRNA quantity of CLEC4F and CD163 mRNA in the livers from wild-type mice and IFNAR1^-/-^ mice at indicated time points (n = 5/each group). (A and B) Data are pooled from two independent experiments and shown as mean ± SEM. The *p*-values were calculated using a one-way ANOVA with Tukey’s multiple comparison test (A) and two-way ANOVA with Tukey’s multiple comparisons test and (B). ns, not significant, ***p < 0.001, ****p < 0.0001.
(PDF)

S2 Fig LysM promoter-mediated Cre recombinase expression. Representative immunostaining images of the livers from reporter mice harboring tdTomato gene under loxP-flanked STOP cassette with or without LysM-mediated Cre recombinase. Scale bars, 100 μm.
(PDF)

S3 Fig HAZV infection induces robust inflammatory responses and loss of Kupffer cells in IFNAR1^fl/fl^ LysM-Cre+ mice. (A) qRT-PCR analysis for relative RNA quantity of HAZV N in the livers from uninfected IFNAR1^fl/fl^ or HAZV-infected IFNAR1^fl/fl^, IFNAR1^fl/fl^ ALB-Cre+, IFNAR1^fl/fl^ LysM-Cre+ and IFNAR1^-/-^ mice at the indicated time points (n = 5/each group). (B) qRT-PCR analysis for relative mRNA quantity of IL-6, TNF-α, and IL-1β mRNA in the livers from uninfected IFNAR1^fl/fl^ or HAZV-infected IFNAR1^fl/fl^, IFNAR1^fl/fl^ ALB-Cre+, IFNAR1^fl/fl^ LysM-Cre+ and IFNAR1^-/-^ mice at 3 dpi (n = 5/each group). (C) qRT-PCR analysis for relative mRNA quantity of CLEC4F and CD163 mRNA in the livers from uninfected IFNAR1^fl/fl^ or HAZV-infected IFNAR1^fl/fl^, IFNAR1^fl/fl^ ALB-Cre+, IFNAR1^fl/fl^ LysM-Cre+ and IFNAR1^-/-^ mice at 3 dpi (n = 5/each group). (A-C) Data are shown as mean ± SEM. The *p*-values were calculated using a one-way ANOVA with Tukey’s multiple comparison test. ns, not significant, *p < 0.05, **p < 0.01, ****p < 0.0001.
(PDF)

S4 Fig IFNAR1^fl/fl^ CX3CR1-Cre+ mice develop lethal disease following HAZV infection. (A, B) Weight change (A) or survival (B) of IFNAR1^fl/fl^ (n = 2) and IFNAR1^fl/fl^ CX3CR1-Cre+ mice (n = 3) following HAZV infection. Arrows indicate the time point at which mice were euthanized for sampling. (C) Virus loads of HAZV in the blood from IFNAR1^fl/fl^ (n = 2) and IFNAR1^fl/fl^ CX3CR1-Cre+ mice (n = 2) at 3 dpi. The detection limit is indicated as a dotted line. (A, C) Data are shown as mean ± SEM. The *p*-values were calculated using a two-tailed unpaired t test. **p < 0.01.
(PDF)
