# Supplementary material for: Loss of type I interferon signaling in Kupffer cells triggers viral spread and fatal liver damage in a mouse model of Crimean-Congo hemorrhagic fever
Source: PLoS Pathog. 2026 Aug 18;22(8):e1014511. doi: 10.1371/journal.ppat.1014511 (PMC13577490; doi:10.1371/journal.ppat.1014511)
Supplement: Supplementary file 1 [file ppat.1014511.s001.pdf]

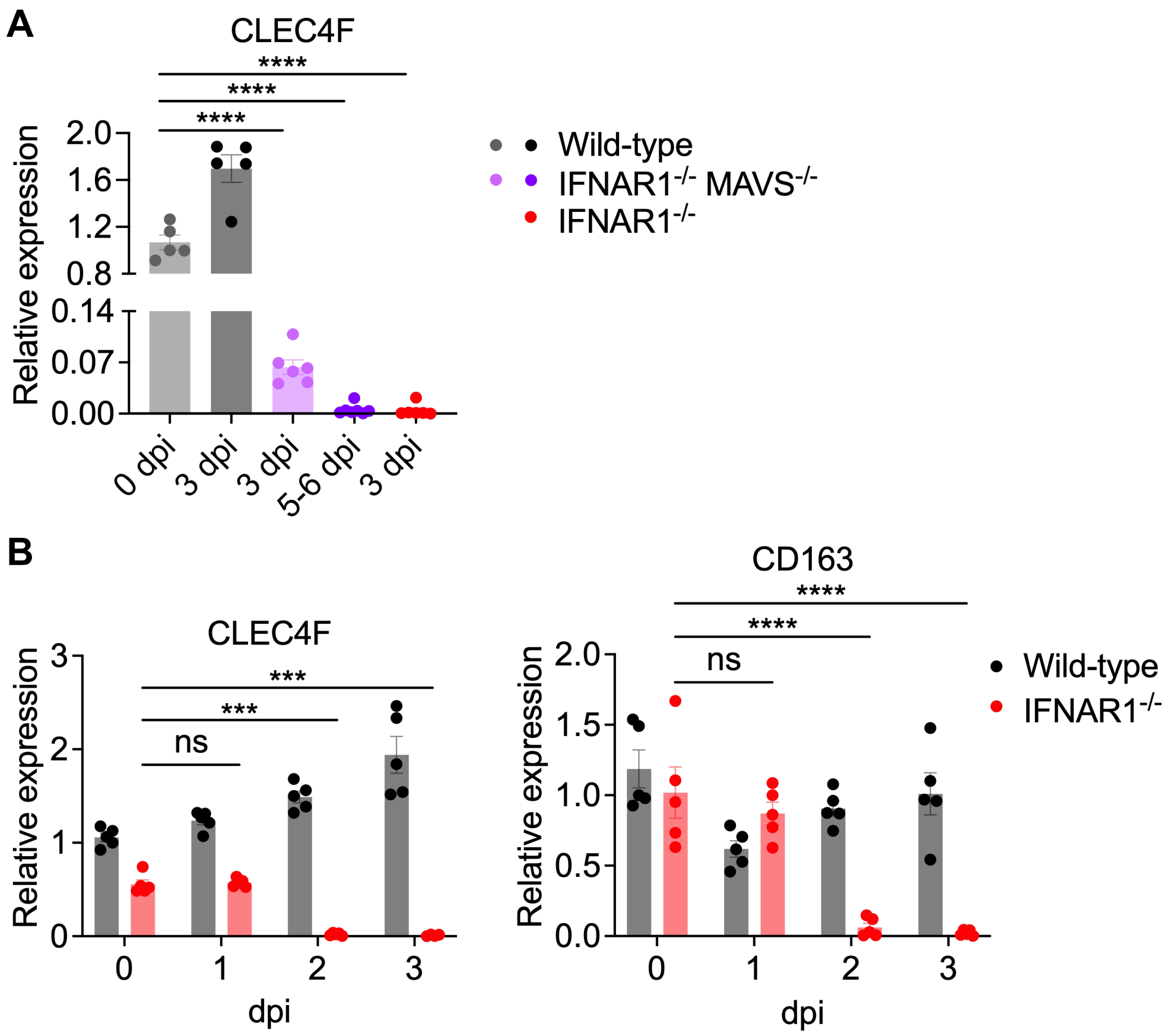

S1 Fig. HAZV infection reduces the expression of Kupffer cell marker genes.

(A) qRT-PCR analysis for relative mRNA quantity of CLEC4F mRNA in the livers from uninfected wild-type mice (n = 5) or HAZV-infected wild-type (n = 5), IFNAR1$^{-/-}$ MAVS$^{-/-}$ mice (n = 6 at 3 dpi, n = 7 at 5-6 dpi) and IFNAR1$^{-/-}$ mice (n = 6) at indicated time points. (B) qRT-PCR analysis for relative mRNA quantity of CLEC4F and CD163 mRNA in the livers from wild-type mice and IFNAR1$^{-/-}$ mice at indicated time points (n = 5/each group). (A and B) Data are pooled from two independent experiments and shown as mean ± SEM. The *p*-values were calculated using a one-way ANOVA with Tukey's multiple comparison test (A) and two-way ANOVA with Tukey's multiple comparisons test and (B). ns, not significant, ***$p < 0.001$, ****$p < 0.0001$.
