# Supplementary material for: Loss of type I interferon signaling in Kupffer cells triggers viral spread and fatal liver damage in a mouse model of Crimean-Congo hemorrhagic fever
Source: PLoS Pathog. 2026 Aug 18;22(8):e1014511. doi: 10.1371/journal.ppat.1014511 (PMC13577490; doi:10.1371/journal.ppat.1014511)
Supplement: Supplementary file 2 [file ppat.1014511.s002.pdf]

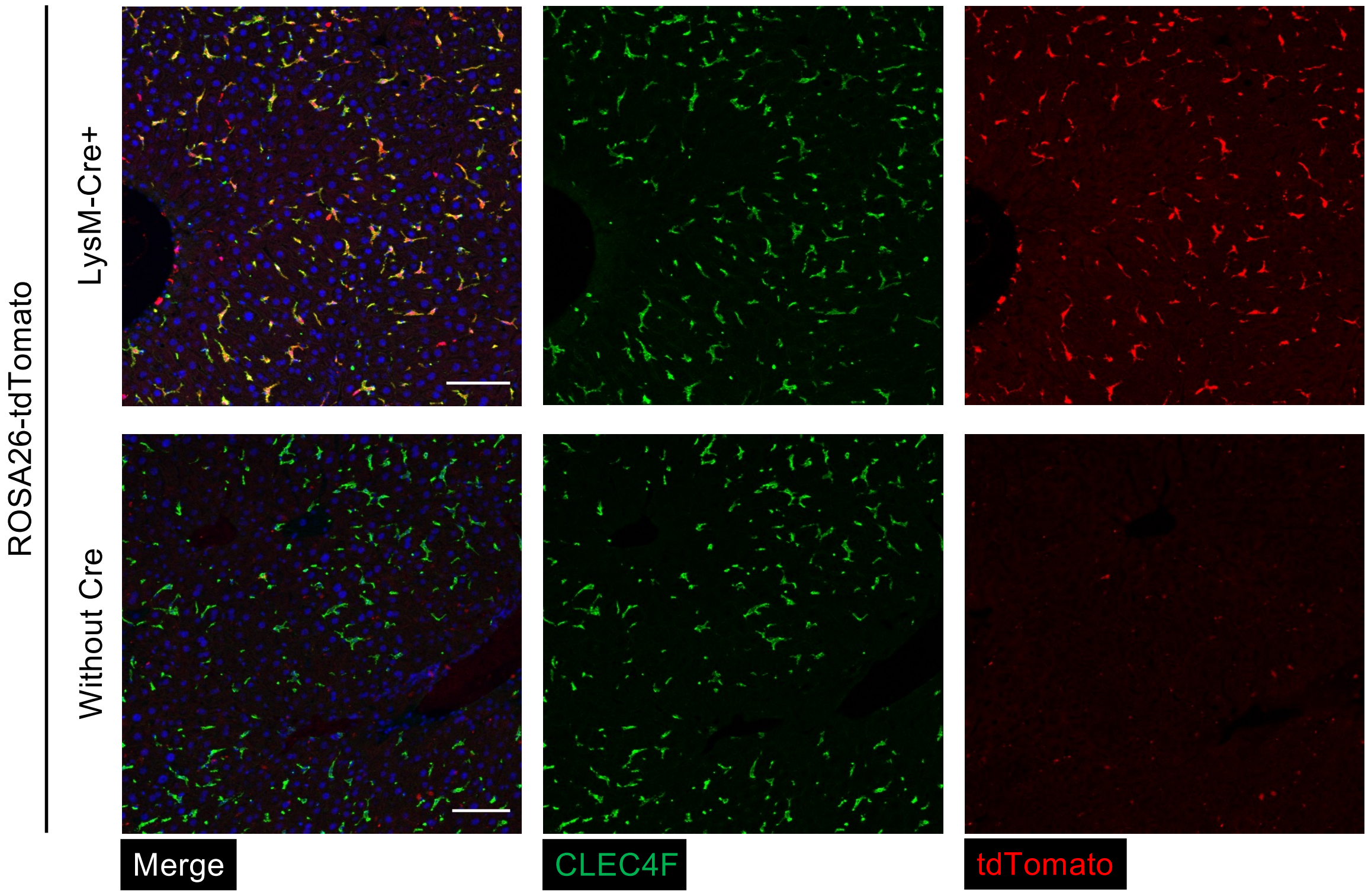

S2 Fig. LysM promoter-mediated Cre recombinase expression.
Representative immunostaining images of the livers from reporter mice harboring tdTomato gene under loxP-flanked STOP cassette with or without LysM-mediated Cre recombinase. Scale bars, 100 μm.
