# Supplementary material for: Loss of type I interferon signaling in Kupffer cells triggers viral spread and fatal liver damage in a mouse model of Crimean-Congo hemorrhagic fever
Source: PLoS Pathog. 2026 Aug 18;22(8):e1014511. doi: 10.1371/journal.ppat.1014511 (PMC13577490; doi:10.1371/journal.ppat.1014511)
Supplement: Supplementary file 3 [file ppat.1014511.s003.pdf]

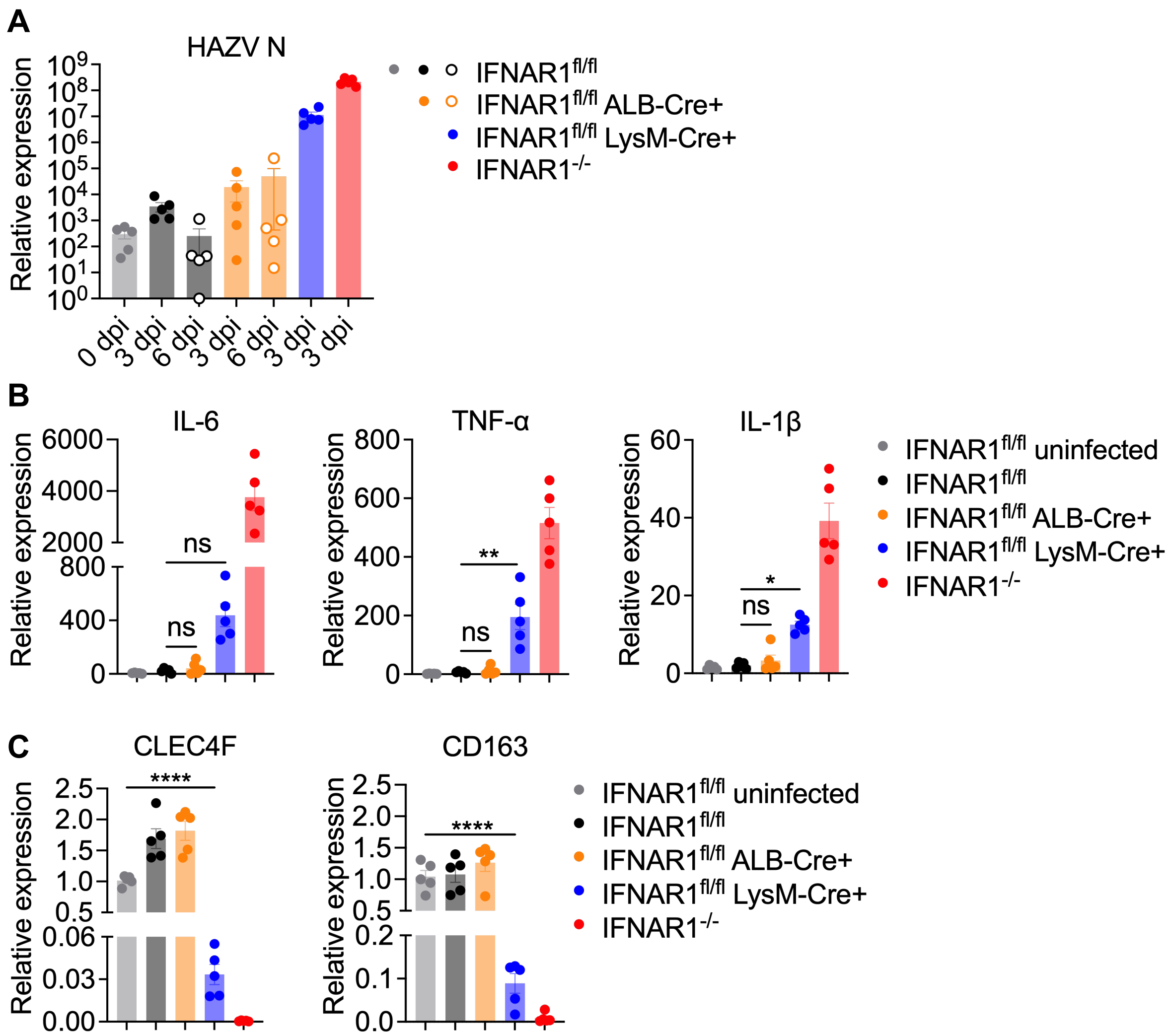


**S3 Fig. HAZV infection induces robust inflammatory responses and loss of Kupffer cells in IFNAR1$^{fl/fl}$ LysM-Cre+ mice.**
(A) qRT-PCR analysis for relative RNA quantity of HAZV N in the livers from uninfected IFNAR1$^{fl/fl}$ or HAZV-infected IFNAR1$^{fl/fl}$, IFNAR1$^{fl/fl}$ ALB-Cre+, IFNAR1$^{fl/fl}$ LysM-Cre+ and IFNAR1$^{-/-}$ mice at the indicated time points (n = 5/each group). (B) qRT-PCR analysis for relative mRNA quantity of IL-6, TNF-α, and IL-1β mRNA in the livers from uninfected IFNAR1$^{fl/fl}$ or HAZV-infected IFNAR1$^{fl/fl}$, IFNAR1$^{fl/fl}$ ALB-Cre+, IFNAR1$^{fl/fl}$ LysM-Cre+ and IFNAR1$^{-/-}$ mice at 3 dpi (n = 5/each group). (C) qRT-PCR analysis for relative mRNA quantity of CLEC4F and CD163 mRNA in the livers from uninfected IFNAR1$^{fl/fl}$ or HAZV-infected IFNAR1$^{fl/fl}$, IFNAR1$^{fl/fl}$ ALB-Cre+, IFNAR1$^{fl/fl}$ LysM-Cre+ and IFNAR1$^{-/-}$ mice at 3 dpi (n = 5/each group). (A-C) Data are shown as mean ± SEM. The *p*-values were calculated using a one-way ANOVA with Tukey's multiple comparison test. ns, not significant, *$p < 0.05$, **$p < 0.01$, ****$p < 0.0001$.
