# Supplementary material for: Loss of type I interferon signaling in Kupffer cells triggers viral spread and fatal liver damage in a mouse model of Crimean-Congo hemorrhagic fever
Source: PLoS Pathog. 2026 Aug 18;22(8):e1014511. doi: 10.1371/journal.ppat.1014511 (PMC13577490; doi:10.1371/journal.ppat.1014511)
Supplement: Supplementary file 4 [file ppat.1014511.s004.pdf]

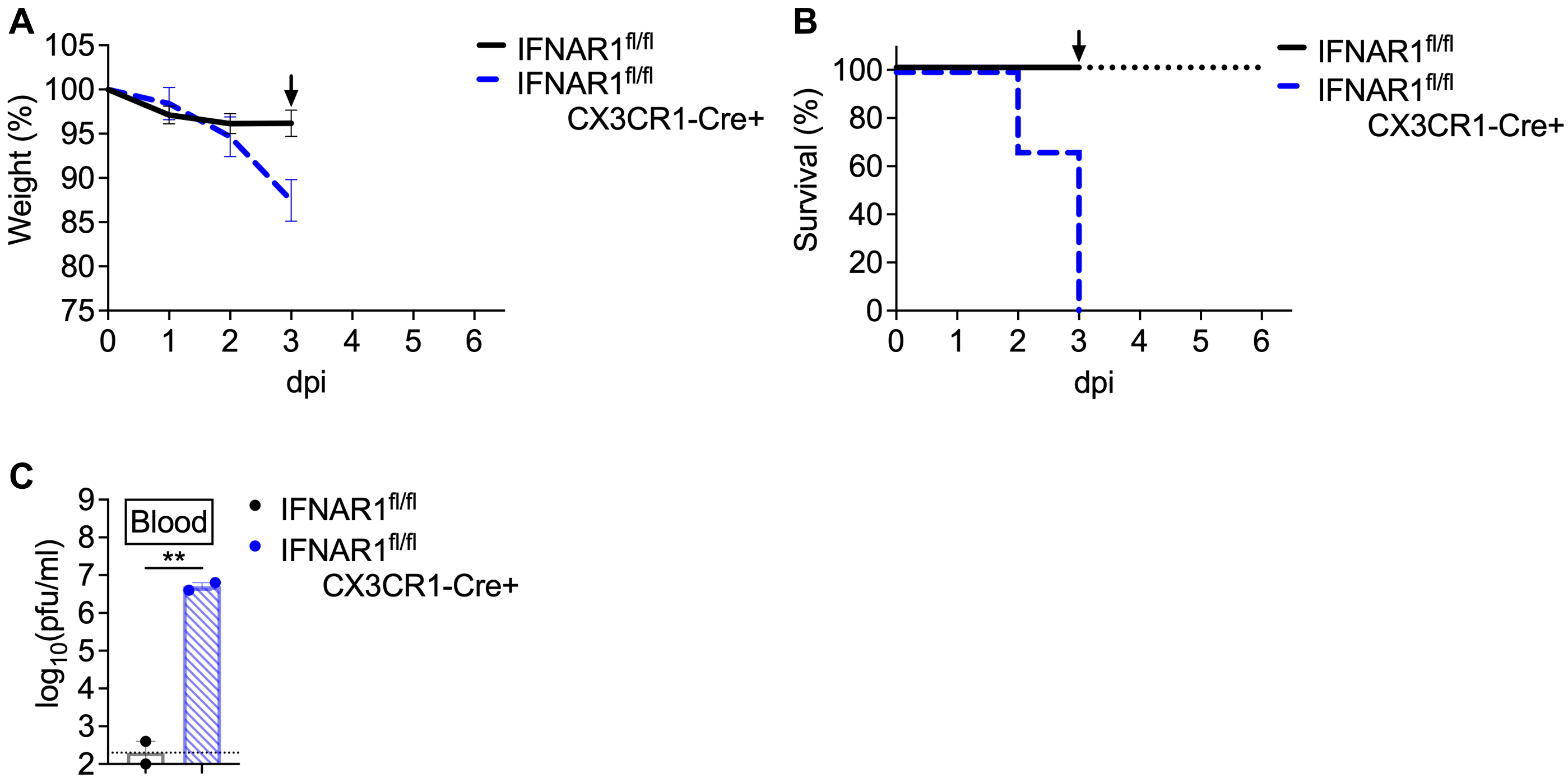

S4 Fig. IFNAR1$^{fl/fl}$ CX3CR1-Cre+ mice develop lethal disease following HAZV infection. (A, B) Weight change (A) or survival (B) of IFNAR1$^{fl/fl}$ (n = 2) and IFNAR1$^{fl/fl}$ CX3CR1-Cre+ mice (n = 3) following HAZV infection. Arrows indicate the time point at which mice were euthanized for sampling. (C) Virus loads of HAZV in the blood from IFNAR1$^{fl/fl}$ (n = 2) and IFNAR1$^{fl/fl}$ CX3CR1-Cre+ mice (n = 2) at 3 dpi. The detection limit is indicated as a dotted line. (A, C) Data are shown as mean ± SEM. The *p*-values were calculated using a two-tailed unpaired t test. ***p* < 0.01.
